# Exercise during pregnancy on maternal lipids: a secondary analysis of randomized controlled trial

**DOI:** 10.1186/s12884-017-1571-6

**Published:** 2017-11-28

**Authors:** Robinson Ramírez-Vélez, Felipe Lobelo, Ana C. Aguilar-de Plata, Mikel Izquierdo, Antonio García-Hermoso

**Affiliations:** 1 Centro de Estudios para la Medición de la Actividad Física (CEMA), Escuela de Medicina y Ciencias de la Salud, Universidad del Rosario, Cra. 24 No. 63C - 69, Bogotá, D.C Colombia; 2 Hubert Department of Global Health, Rollins School of Public Health, Emory University, Atlanta, GA USA; 3 Nutrition Group, Universidad del Valle, Cali, Colombia; 4 Department of Health Sciences, Public University of Navarre, CIBER de Fragilidad y Envejecimiento Saludable (CB16/10/00315), Pamplona, Navarra Spain; 5 Laboratorio de Ciencias de la Actividad Física, el Deporte y la Salud, Facultad de Ciencias Médicas, Universidad de Santiago de Chile, USACH, Santiago, Chile

**Keywords:** Physical activity, Prenatal, Metabolic biomarkers, Obstetric outcomes

## Abstract

**Background:**

Today, scientific evidence has supported the popular belief that physical activity is associated with biological health in pregnant women. A randomized controlled trial was used to assess the benefits of physical exercise during pregnancy on maternal lipids in low-income Latina women.

**Methods:**

The study included 67 nulliparous low-income Latina women in gestational weeks 16–20, randomly assigned into one of two groups: 1) The exercise group, which took part in aerobic and resistance exercise for 60 min, three times a week for 12 weeks, 2) The control group, which undertook their usual physical activity and prenatal care. The primary outcomes were changes in maternal blood lipids after intervention. Obstetrical and neonatal outcomes measured were type of delivery, postpartum hemorrhage, newborn and/or maternal complications’, gestational age, weight gain, birth weight, foetal growth, and Apgar score.

**Results:**

Fifty women completed the study. At the end of the intervention, there were differences between groups in low-density lipoprotein levels (mean change: −8 mg/dL, 95%CI -3 to −29; *P* < 0.001) and triglycerides (mean change: −6 mg/dL, 95%CI -1 to −11; *P* = 0.03). Also, compared with women who remained in the control group, active women showed lower complications during delivery (moderate postpartum haemorrhage) (58% compared with 75%; *P* < 0.05) and lower complications in newborns (e.g. cyanosis or respiratory distress) (21% compared with 46%; *P* < 0.001).

**Conclusions:**

An exercise programme during the second and third trimester favours less gain in low-density lipoprotein cholesterol and triglycerides fewer delivery and neonatal complications.

**Trial registration:**

NCT00741312 (August 22, 2008).

## Background

During pregnancy, regular physical activity is associated with the course of a healthy pregnancy, as it can increase physical fitness, and may lower the risk of pre-eclampsia, gestational diabetes, chronic hypertension, excessive gestational weight gain, macrosomia, and stillbirth [1, 2]. Guidelines from the American College of Obstetricians and Gynecologists (ACOG) [3] recommend regular exercise for pregnant women, including those who are sedentary, for its overall health benefits in maternal and neonatal outcomes [4–6]. Yet few pregnant women achieve an appropriate level of physical activity, in part because they are uncertain about the types and amount of exercise that can and should be performed [7].

For example, African-American and Latina women have higher risk of developing several complications, such as gestational diabetes mellitus (GDM), chronic hypertension, pre-eclampsia and Caesarean delivery in comparison to non-Latina white and non-obese women [8, 9]. GDM in Latina women is two to three times more prevalent than in non-Latinas. Additionally, foetal and neonatal deaths due to diabetes and pregnancy are three to eight times more prevalent in pregnancies of diabetic mothers, and there is a greater risk for congenital malformations in children [8]. Latina women with other environment conditions such as lower socio-economic status or educational attainment, history of physical inactivity prior to pregnancy, lack of social support, and lower employment status are at higher risk for developing several maternal and neonatal complications [9]. Clinicians are hesitant to advise sedentary women to initiate supervised physical exercise during pregnancy, due to the possibility of exercise-induced risk of preterm delivery or foetal stress [10].

Despite evidence that exercise improves lipid profiles in non-pregnant populations, in pregnant women this evidence is scarce. Dyslipidaemia during pregnancy is associated with GDM, pre-eclampsia, preterm birth [11] and other adverse outcomes such as preterm delivery [12], low birth weight [13], and risk of macrosomia [14].

Recent results support an effect of supervised physical exercise on triglyceride (TG) levels during pregnancy [15, 16], however findings with other lipids appear to be less consistent [17]. While the major causes remain unknown, these controversial results could be explained by differences in trimester of pregnancy, exercise programmes, and nutrition [7].

Therefore, the purpose of this prospective, randomized study was to assess the benefits of supervised physical exercise during pregnancy, using a recommended physical activity dose for maternal lipids in low-income pregnant Latina women. Secondary aims were to assess the effect of exercise on obstetrical and neonatal outcomes.

## Methods

### Design overview

The present study is a secondary analysis randomized controlled trial (RCT) published previously [18, 19]. The RCT was a randomized trial conducted from March 2008 to January 2010. Briefly, 67 participants were recruited at three prenatal care outpatient clinics in Cali, Colombia (Hospital Cañaveralejo, Centro de Salud Siloe or Centro de Salud Melendez). After confirmation of eligibility, the women were assigned randomly to the following groups: i) aerobic and resistance exercise training plus usual prenatal care, or ii) usual care only. All investigators received training before the trial concerning the protocol and assessments.

Measurements were taken at baseline for participant women in their first trimester of gestation (all between gestational weeks 16–20) and at the end of the exercise intervention period when participant women were in their third trimester of gestation (corresponding to gestational weeks 28–32) [18, 19]. All protocols followed were in accordance with ethical standards of research and the Helsinki declaration and participants received written information about the study (potential sources of risk and benefits). In the case of participants under 18, this information was provided to their parents/guardians. All participants and the parents/legal guardians of minors under 18 gave their informed written consent before the study began. The participants were not compensated financially but were provided food (light breakfast) before each exercise session. Finally, the RCT was approved by the Committee for Medical Research Ethics (UV Res. 004/08; N°142–07) and was registered with clinicaltrial.gov [NCT00741312].

### Participants

Inclusion criteria were: i) women aged between 16 and 30 years; ii) being physically inactive (<150 min·wk.^−1^ of moderate-intensity activity or 75 min·wk.^−1^ of vigorous-intensity activity); iii) nulliparous; iv) were in their 16th to 20th week of gestation; and v) with a live foetus confirmed by a routine ultrasound scan [18, 19].

### Exercise intervention

Women in the intervention group participated in three 60-min supervised exercise sessions per week for 12 weeks. Exercise training sessions were designed to elicit a response in the acceptable moderate-to-vigorous intensity at 55–75% of maximal heart rate (HR). In addition, intensity was adjusted according to ratings on the modified Borg scale, using a rate of perceived exertion ranging from 4 to 7 [20, 21]. Sessions consisted of a warm-up walk (10 min) followed by an aerobic exercise session (30 min), resistance exercise (10 min), and a final relaxation/cool-down period (10 min). At each exercise session (rest, 15 and 30 min), participants wore an HR monitor (Polar Pacer, USA) to ensure compliance with the exercise stimulus at the predetermined target HR zone. Systolic and diastolic blood pressure were also analysed in each exercise class using a manual aneroid sphygmomanometer by Riester (Jungingen, Germany) and a 3 M Littmann stethoscope (3 M Health Care, St Paul, MN, USA). Detailed descriptions of each exercise station have been previously published [18, 19].

Each woman carried out an individualized nutrition intervention plan devised by a dietician [18, 19]. Every training, each woman received a light breakfast/meal 45 min before the exercise session following the specific recommendations during pregnancy (approximately (400 kcal) [up to 40–55% carbohydrates, up to 30% fat and up to 20–30% protein) [22, 23]. The control group received standard prenatal care (1 session per week for 12 weeks) with no exercise intervention or light breakfast/meal.

### Primary outcome

One day before beginning and immediately after the 12-week exercise programme participants were invited for two measurement sessions. Ten millilitres of blood were drawn from the antecubital vein into Vacutainer tubes with no additives. Metabolic biomarkers were measured using the following procedures: TG, and high-density lipoprotein cholesterol (HDL-c) were tested using a direct colorimetric method in an automated spectrophotometer (Biosystems, Spain) [24]. Very-low density lipoprotein cholesterol (VLDL-c) and low-density lipoprotein cholesterol (LDL-c) were calculated using the Friedewald et al. equations [25].

### Secondary outcomes

Anthropometric and adiposity variables: body weight was measured to the nearest 0.10 kg with the participant lightly dressed using a portable electronic weight scale (Webb City, MO, USA) within 0.1 kg of precision. Body height was measured to the nearest 0.1 cm in bare or stocking feet, with the participant standing upright against a portable stadiometer (Seca® 274, Hamburg, Germany. Their BMI was calculated as their body weight in kilograms divided by the square of their height in metres.

Pregnancy complications: Obstetrical and medical complications during delivery (i.e.: postpartum hemorrhage) were recorded by midwives or obstetricians upon delivery.

Delivery data: The following data were recorded: i) Gestational age in weeks and days from hospital perinatal records; ii) the type of delivery; iii) weight gain in gr; iv) Apgar scores at 1 and 5 min and newborn complications (i.e. appearance [skin colour], pulse [heart rate], grimace [reflex irritability], activity [muscle tone], and respiration test [range 0 to 10]) [26].

Anthropometric assessments of newborns: 60 min after delivery birth weight (SECA scale ±10 g), crown–heel length, head circumference, chest circumference (Cambridge Scientific Instruments, Cambridge, MD) were recorded using standard methods by a hospital nurse.

### Data analysis

An exploratory analysis using the *Kolmogorov–Smirnov* test was first performed to assess the normality of the distribution for each variable. Primary data analysis was performed using the intention-to-treat principle (patients who did not complete the intervention were also included) to evaluate differences in maternal outcomes by intervention groups, including their baseline measurement and the intervention time as co-variables. The combined effect of the exercise training programme duration and intervention status was explored. The interaction between groups (exercise and control) and time (pre- and post-test) was used to calculate the baseline-adjusted differences between groups. Neonatal and obstetric outcomes were compared using the *t-test* or *Mann–Whitney U-*test according to the normality of the variables. A bivariate analysis was carried out in which categorical variables were compared using the *chi-square* test or *Fisher* test, as appropriate. All the analyses were performed using SPSS software (version 17.0) and the level of significance was set at *p* < 0.05.

Sample size calculation: Data from a pilot study disclosed that 30 participants per group were adequate to detect a 10% difference in TG level (p < 0.05 level and a power of 0.80) (Power and Sample Size Calculation, Los Angeles, CA).

## Results

### Flow of participants and baseline characteristics

**Fig. 1:**
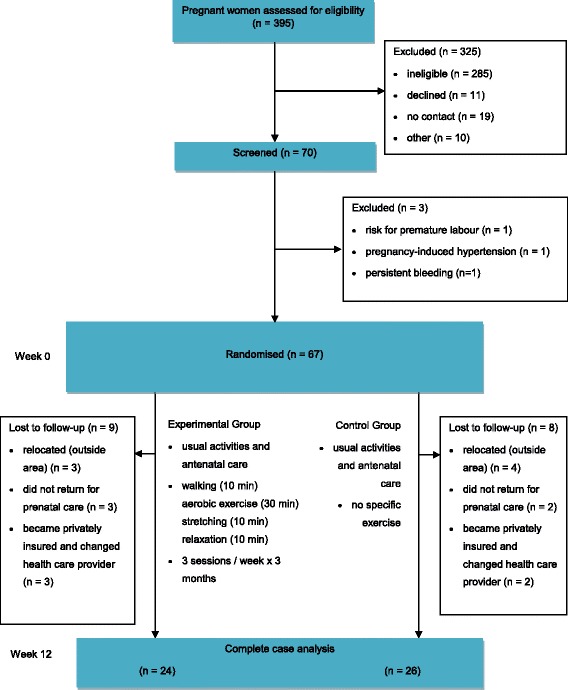
Design and flow of participants through the study

Characteristics of the women are presented in Table 1. Thirty-three women were allocated to the experimental group and 34 to the control group. At the end of the intervention, there were 24 participants in the experimental group and 26 in the control group. Fig. 1 shows the flow of women through the study.

**Table 1:** Baseline characteristics of participants by study completion and Intervention status

Characteristic	Study completers(*n* = 67)		Lost to follow-up(*n* = 13)	
	Intervention(*n* = 33)	Control(*n* = 34)	Intervention(*n* = 9)	Control(*n* = 8)
Participants, mean (SD)				
Age*,* years	19 (3)	20 (3)	19 (2)	19 (2)
Gestation*,* week	18 (3)	17 (4)	18 (2)	19 (3)
Socioeconomic level, *n* (%)				
Low-mid	30 (91)	31 (91)	2 (22)	2 (25)
Mid-high	3 (9)	3 (9)	7 (88)	6 (75)
Education, *n* (%)				
None/Primary	7 (21)	10 (30)	4 (44)	2 (25)
Secondary	20 (61)	21 (62)	2 (22)	2 (25)
Technical/University	6 (18)	3 (9)	3 (33)	4 (51)
Occupation, *n* (%)				
Student	10 (30)	7 (21)	3 (33)	3 (38)
Housewife	23 (70)	27 (79)	6 (67)	5 (63)
Residence Location, *n* (%)				
Urban	29 (88)	27 (79)	3 (33)	3 (38)
Rural	4 (12)	7 (21)	6 (67)	5 (63)

Data are mean (SD) or n (%)

### Compliance

**Fig. 2:**
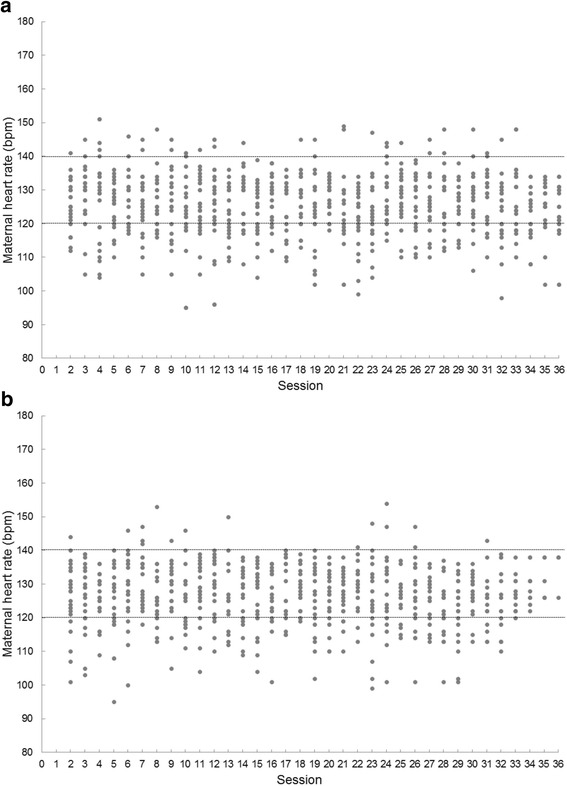
Maternal heart rate changes during moderate-intensity exercise sessions according with currently recommended aerobic physical activity guidelines during pregnancy. Average intensity based on a target heart-rate zone of 120 to 140 bpm, according with currently recommended aerobic physical activity guidelines during pregnancy. Experimental participants received on average 28.9 out of 36 (SD 3.2) sessions over the 12 weeks. No adverse events occurred during or after the exercise in any participant. (**a**) First 15 min 16, (**b**) 15–40 min of exercise

Nine women in the experimental group and eight in the control group withdrew from the study before the intervention measurements. The reasons were the following: i) scheduling conflicts; ii) employment hours; and iii) transportation issues contributed to most dropouts. The 24 active women from the exercise group participated in 28.9 out of 36 (SD 3.2) sessions over the 12 weeks without adverse events. During the exercise sessions it was observed that in 15% (122 sessions) of the 864 scheduled sessions, participants in the intervention remained at an exercise intensity below the minimum scheduled dose after 15 min (Fig. 2a), while 11% (93 sessions) of participants remained below the minimum after 30 min of exercise (Fig. 2b).

### Effect of intervention

#### Primary outcomes

At the end of the intervention programme, the multivariable analysis, which we adjusted for baseline levels, showed a difference between groups in LDL-c (-8 mg/dL, 95%CI -3 to −29; *P* < 0.001) and TG (−6 mg/dL, 95%CI 1 to 11; *P* = 0.03).

#### Secondary outcomes

There were no significant differences between the groups regarding maternal weight gain and BMI during the exercise programme period, Table 2.

**Table 2:** Effect of the exercise training program on maternal anthropometric and metabolic biomarkers by intervention and control groups adjusted for baseline values

	Groups				Delta between groups	*P*
	Baseline		Follow-up		Follow-up minus Baseline	
	Intervention(*n* = 33)	Control(*n* = 34)	Intervention(*n* = 24)	Control(*n* = 26)	Difference between groups (95% CI)	
Anthropometric						
Weight, kg	53.3 (6.1)	55.8 (8.0)	61.1 (5.1)	63.0 (7.2)	0.6 (−4.0 to 2.0)	0.82
BMI, kg/m^2^	21.8 (2.4)	23.5 (3.1)	25.2 (1.8)	26.3 (3.1)	0.6 (−0.2 to 1.4)	0.91
Metabolic biomarkers						
Total cholesterol, mg/dL	198 (52)	178 (39)	249 (40)	236 (49)	-6 (−21 to 33)	0.45
High-density lipoprotein, mg/dL	50 (11)	53 (13)	57 (10)	67 (14)	−8 (1 to 17)	0.16
Low-density lipoprotein, mg/dL	81 (38)	63 (25)	78 (17)	73 (23)	−13 (−3 to 29)	<0.001
Very-low density lipoprotein, mg/dL	28 (12)	23 (7)	39 (9)	40 (8)	−6 (1 to 11)	0.50
Triglycerides, mg/dL	139 (62)	116 (37)	195 (45)	199 (42)	−28 (1 to 55)	0.03

Data are mean (SD)

There was no significant difference in gestational age or early postnatal measures (head circumference, chest circumference, crown–heel length and Apgar score) between groups (Table 3). In the same vein, the percentage of preterm deliveries did not differ between the exercise and control group (*P* = 0.64). Two and three women showed preterm delivery in the experimental and control group, respectively. There was no significant difference between the experimental and control groups regarding mean birth weight (3133 ± 406 g versus 3013 ± 494 g, *P* = 0.34), low birth weight (< 2500 g; (*n* = 3 versus *n* = 2) or high birth weight (> 4000 g; *n* = 1 versus *n* = 0). In both groups the same medical reasons for low weight were observed in the mother [HELLP syndrome, pre-eclampsia, hypertension, and oligohydramnios], (33% compared with 27%, *P* = 0.80). The experimental group showed a higher percentage in of Caesarean sections in comparison to the control group without significant differences (27% compared with 13%, *P* = 0.89). The experimental group showed fewer complications during delivery (postpartum hemorrhage moderate) than did the control group (58% compared with 75%, *P* = 0.05). Finally, the experimental group showed fewer complications in newborns compared to the control group (21% compared with 46%, *P* = 0.01) (Table 3).

**Table 3:** Effect of the exercise-training program on obstetrical and neonatal outcomes

Outcome	Group		*P*
	Intervention(*n* = 24)	Control(*n* = 26)	
Gestational age at delivery, days	38.9 (2.2)	39 (1.8)	0.36
% of preterm deliveries (37 complete weeks) by the end of the study period, *n* (%)	2 (8)	3 (13)	0.64
Early postnatal measures			
Birth weight (g)	3133 (406)	3013 (494)	0.34
Low birth weight (<2500 g),* n* (%)	3 (12)	2 (10)	0.75
High birth weight (>4000 g),* n* (%)	1 (4.1)	0	0.78
Head circumference (cm)	33.7 (1.6)	32.8 (2.7)	0.47
Chest circumference (cm)	32.6 (1.8)	32.4 (2.0)	0.98
Crown-to-heel length (cm)	50.5 (2.4)	50.1 (2.2)	0.63
APGAR score (1 min)^a^	8 (7–9)	8 (7–9)	0.36
APGAR score (5 min)^a^	9.5 (9–10)	10 (9–10)	0.25
Sex (newborn)			
Male, *n* (%)	11 (46)	13 (50)	0.65
Type of delivery			
Caesarean section, *n* (%)	3 (13)	7 (27)	0.89
Postpartum hemorrhage			
Low-to Moderate, *n* (%)	14 (58)	22 (75)	0.01
Maternal complications			
HELLP syndrome, preeclampsia *or* oligohydramnios, *n* (%)	8 (33)	7 (27)	0.80
Newborn complications			
Meconium, cyanosis *or* respiratory distress, *n* (%)	5 (21)	12 (46)	0.01

Continuous variables (presented as means [SDs]) were analyzed using t-test *or* ^a^Mann–Whitney *U*-test (presented as median [interquartile range]) according to the normality of the variables. Categorical variables (presented as n values [percentages]) were analyzed using chi-square test or fisher test

## Discussion

Our study shows that physical exercise reduces the c-LDL and TG increases in normal ranges, favouring fewer delivery and neonatal complications. Beneficial relations between physical activity and the plasma lipid profile in non-pregnant women have been demonstrated. Findings reported that the sensitivity of the regression slopes for HDL and TG imply that women may be more resistant to exercise-induced changes than men [27]. A meta-analysis from 145 longitudinal studies suggested that exercise training, especially for those at risk for heart diseases (elevated pre-exercise cholesterol concentrations) is beneficial to the lipid profile of women [28]. In pregnant women, there are few studies that have analysed these relations during pregnancy.

Data from the OMEGA study reported that active women (early in pregnancy) had significantly lower mean TC and TG in comparison to those of women performing no recreational physical activity [29]. Other studies suggest a positive effect of physical activity on TG levels during pregnancy [15, 16], however results with other lipids are scarce. For example, data from NHANES (2003–2006) found that sedentary behaviour, assessed by accelerometry, was associated with higher LDL-c levels and moderate to vigorous physical activity with higher HDL-c levels in pregnant women [16].

Our results showed that physical exercise reduces the excessive LDL-c and TG gain. Therefore, findings seem to suggest that early pregnancy physical activity could have a lasting impact on TG levels. In contrast, another study showed, surprisingly, that the exercise group had significantly higher LDL-c levels in the third trimester in comparison to the control group [17]. These findings could be due to the fact that after intervention, the exercise group had significant only moderate differences in the physical activity levels; also, the intensity of the exercise was not reported, therefore we cannot make comparisons with our exercise programme.

A meta-analysis from 11 RCTs in sedentary women provides evidence that moderate-intensity physical exercise during physical activity in pregnancy does seem to positively influence excessive gestational weight gain [30]. Another recent meta-analysis confirmed that diet- and physical activity-based interventions during pregnancy reduce gestational weight gain, but physical activity alone offers similar effects as mixed interventions [7]. However, it needs to be taken into account that weight gain in our study was assessed after 12 weeks of the exercise and not previous to delivery as in other studies.

Regarding obstetrical and neonatal outcomes, our study showed that exercise performed over the second and third trimesters of pregnancy does not negatively influence gestational age, Caesarean delivery or neonatal growth. Previous studies investigating the effect of exercise during pregnancy and birth weight report inconsistent findings [31]. Several pilot non-controlled studies [32] and RCTs [33, 34] show that exercise during pregnancy could be beneficial overall to the maternal–foetal unit. Also, prospective studies suggest no significant association between physical activity during pregnancy and pregnancy outcome in active women [32].

A few limitations in this study should be considered and caution should be taken in the interpretation of the findings. Firstly, due to withdrawals, this study was underpowered to detect differential effects of supervised exercise training on blood lipids. Secondly, there was no assessment of other biomarkers or dietary nutrition intake, which could have shown additional information on metabolic health status. Thirdly, therapists and women were not blinded. However, comparison of the final study population with the women who did not complete the study did not identify any differences between groups. The pregnant participants in our study were healthy nulliparous with a low-income level, and are therefore not representative for all eligible Latina women.

The strengths of our study include the consideration of important confounding variables in our analysis, such as compliance and obstetrical and neonatal outcomes. First, physical activity recommendations during pregnancy were evaluated. Second, women underwent a peak exercise test to more accurately prescribe the exercise intensities.

## Conclusion

In summary, exercise during the second and third trimester of pregnancy in sedentary women reduced excessive LDL-c and TG gain and favoured fewer delivery and neonatal complications without any adverse acute foetal responses to current exercise recommendations. Hence, implementation of an exercise-training programme is feasible and safe in low-income Latina women. The potential public health benefits of exercise are too great for obstetricians to miss the opportunity to effectively counsel pregnant low-income Latina women about this important health behaviour.

## Abbreviations

AGOC: Guidelines from the American College of Obstetricians and Gynecologists
BMI: Body mass index
GDM: Gestational diabetes mellitus
HDL-c: High-density lipoprotein cholesterol
HR: Heart rate
LDL-c: Low-density lipoprotein cholesterol
RCT: Randomised controlled trial
TG: Triglycerides
VLDL-c: Very-low density lipoprotein cholesterol
